# Genotypic Influence on Aversive Conditioning in Honeybees, Using a Novel Thermal Reinforcement Procedure

**DOI:** 10.1371/journal.pone.0097333

**Published:** 2014-05-14

**Authors:** Pierre Junca, Julie Carcaud, Sibyle Moulin, Lionel Garnery, Jean-Christophe Sandoz

**Affiliations:** Evolution, Genomes et Speciation Lab (LEGS – UPR 9034), CNRS, Gif-sur-Yvette, France; Universidade de São paulo, Brazil

## Abstract

In Pavlovian conditioning, animals learn to associate initially neutral stimuli with positive or negative outcomes, leading to appetitive and aversive learning respectively. The honeybee (*Apis mellifera*) is a prominent invertebrate model for studying both versions of olfactory learning and for unraveling the influence of genotype. As a queen bee mates with about 15 males, her worker offspring belong to as many, genetically-different patrilines. While the genetic dependency of appetitive learning is well established in bees, it is not the case for aversive learning, as a robust protocol was only developed recently. In the original conditioning of the sting extension response (SER), bees learn to associate an odor (conditioned stimulus - CS) with an electric shock (unconditioned stimulus - US). This US is however not a natural stimulus for bees, which may represent a potential caveat for dissecting the genetics underlying aversive learning. We thus first tested heat as a potential new US for SER conditioning. We show that thermal stimulation of several sensory structures on the bee’s body triggers the SER, in a temperature-dependent manner. Moreover, heat applied to the antennae, mouthparts or legs is an efficient US for SER conditioning. Then, using microsatellite analysis, we analyzed heat sensitivity and aversive learning performances in ten worker patrilines issued from a naturally inseminated queen. We demonstrate a strong influence of genotype on aversive learning, possibly indicating the existence of a genetic determinism of this capacity. Such determinism could be instrumental for efficient task partitioning within the hive.

## Introduction

To survive, animals must be able to associate stimuli of their environment with their positive or negative consequences. This leads to two complementary forms of associative learning, termed respectively ‘appetitive’ and ‘aversive’ learning. A major question in the study of the neural bases of cognitive functions is the relationship existing between these two types of associative learning [1]–[5]. Strongly related to this question is the search for the genetic architecture underlying these two learning types. Do they rely on utterly different ensembles of genes, giving rise to mostly independent neural processes, or do they share essential characteristics, such as for instance the associative machinery?

In this prospect, honeybees (*Apis mellifera*) may represent a valuable asset. In addition to being a well investigated invertebrate model for the study of the behavioral and neuronal basis of associative learning and memory [6]–[8], the genetic architecture of their colonies is well adapted for studying a possible genotypic influence on cognitive skills. Honeybees possess a haplo-diploid reproduction system. In a honeybee colony, the diploid queen mates on average with fifteen haploid males [9]. Therefore, the workers, her daughters, make up about fifteen different patrilines with different genetic backgrounds within the hive. It is currently thought that such genetic diversity is beneficial for the colony’s fitness and survival [10]. Indeed, post-winter survival rate, production of sexuals, resistance and swarming were found to be positively correlated to the number of patrilines [11]. Moreover, a high number of patrilines results in an increased performance for thermoregulation, food storage, and even worker communication during foraging [12]–[13]. How can these advantages be explained in terms of task allocation within the hive? An important ensemble of theories, named “threshold theories”, consider that the different responsiveness of each individual to environmental stimuli determines this individual’s propensity to engage in one or another behavioral task [12], [14]. Thus, the existence of different patrilines with diversified responsiveness within the hive would allow optimal task allocation, in particular concerning foraging [15]–[16] or thermoregulation [13]. One may thus ask what is the influence of patriline origin on bees’ sensitivity to appetitive and aversive reinforcement and on their learning capacity in these two modalities.

Until now, however, the search for a genetic determinism of associative learning in bees has been limited to appetitive learning, due to the long existence of a well-established laboratory assay: the conditioning of the proboscis extension response (PER) [17]–[18]. The proboscis extension is a reflex triggered by sugar stimulation provided on gustatory receptors of the antennae, tarsi or mouthparts. In olfactory PER conditioning, an originally neutral odor (conditioned stimulus – CS) is associated with a sugar reward first presented to the antennae and then to the proboscis (unconditioned stimulus – US). Once the association has been established, the bee responds with a proboscis extension to the odor (CS) alone. Thanks to this biological assay, a number of studies have evaluated the relative influence of genetic, developmental and environmental factors on appetitive learning and established its genetic dependency [19]–[23]. This dependency relies in part on bees’ responsiveness to the sugar (US), a highly genetically-dependent trait which strongly influences the future role of workers as nectar, pollen or water foragers [24]–[25]. Bees’ responsiveness to sugar directly affects appetitive learning performances [26]–[27]. Bees with a high response threshold perceive the sugar reward as less intensive, and therefore learn it less efficiently than bees with a lower threshold [28]. It seems that many behavioral traits of the honeybee are correlated with sugar responsiveness, as for example olfactory sensitivity and phototactic behavior [29]. As a result, the authors of these studies even suggested that sugar responsiveness could be the *only* determinant of honeybee behavior [25]. However, it was later found that this hypothesis did not take into account types of behaviors that are not related to food search, such as for instance defense behavior or aversive learning [30].

This lack of data on the aversive aspects of honey bee behavior was mainly due to the absence of dedicated protocols for studying aversive learning in controlled laboratory conditions. Recently, the Pavlovian conditioning of the sting extension response (SER) was developed to solve this problem [31], [8]. An electric shock applied to the bee’s thorax triggers an extension of the sting [32]. Bees can learn to associate an odor CS with this electric shock US and after conditioning will respond to the punished odor with a SER [31]. Since then, it was shown that bees which are more sensitive to the electric shock learn and memorize odor-shock associations more efficiently [30]. However, to what extent the observed inter-individual variability in sensitivity to the aversive US and in aversive conditioning capacity relies on a genetic determinism is as yet unknown.

One potential caveat when studying the genetic basis of associative learning could be the unnatural quality of the electric shock as a US. First, the electric shock is applied broadly on the bee’s body, which makes it difficult to know which structure(s) has (have) been stimulated. Second, it is still unclear if the electric shock is detected by particular receptors at the periphery, or if it also acts through direct electric activation of peripheral or more central neurons. Using a more natural aversive US, for which the honeybee has evolved dedicated peripheral receptors and neural pathways, may thus be beneficial for addressing the genetics of aversive learning. We thus first aimed to develop a version of SER conditioning which uses a natural stimulus as US: temperature.

In the honeybee colony, workers maintain a temperature comprised between 32°C and 36°C, mainly because brood development is highly dependent on ambient temperature [33]–[34]. At the individual level, honeybees strictly avoid temperatures above 44°C, and reject sucrose solution presented at 45–50°C [35]. A high temperature is therefore a naturally aversive stimulus for bees. A thermal stimulus can be applied locally, on particular sensory organs of the bee, using small heated copper probes (see Materials and Methods). In addition, some data are already available on the peripheral detection of temperature in honeybees. The antennae, for instance, contain a specific type of sensilla, the *coelocapitular* sensilla, which detect warmth [36]. Moreover, a honeybee-specific thermal receptor, HsTRPA (Hymenoptera specific Transient Receptor Potential Ankyrin) has been recently identified [35]. This receptor is present in many sensory structures, such as the antennae, the proboscis and the legs. However, even if we know that bees actively avoid heat and possess warm sensitive receptors on many of their sensory organs, we do not know if a thermal stimulus can trigger a defensive response of sting extension. We also do not know if this stimulus can play the role of an aversive reinforcement.

The goal of this study was to determine how genotype differences impact aversive olfactory learning in the honey bee, using a natural aversive US. To address this question, we first asked whether local thermal stimulation on the honeybee body can trigger SER. We tested responses to thermal application on the antenna, the mouthparts, the legs and the abdomen, and determined the temperature sensitivity of these structures. Next, we developed a new version of the SER conditioning protocol using a thermal stimulation as US. Then, we compared how sensitivity to temperature and aversive learning performances interact at the individual level. Lastly, we used a genetic analysis based on microsatellites to assess whether a bees’ genotype influences this relationship.

## Results

### Experiment 1: Effect of Temperature on the Sting Extension Response

**Figure 1:**
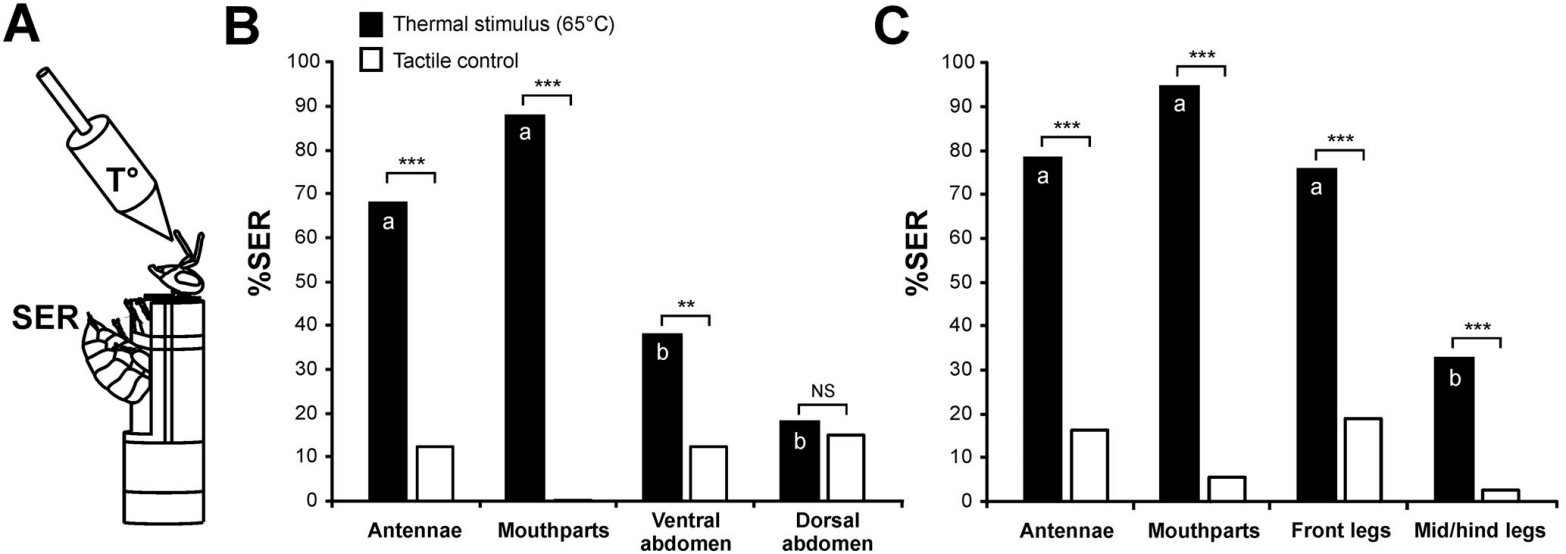
Thermal stimulation on different structures of bee’s body. **A)** Bee harnessed in a conditioning tube, leaving the whole abdomen free and allowing observation of sting extension responses (SER). Thermal stimulations were applied using a heated copper probe. As control, tactile stimulations were applied with an identical unheated probe. **B)** Percentage of SER to 1s thermal stimulations (65°C) and to tactile controls on: the antennae, the mouthparts, the ventral abdomen, the dorsal abdomen (n = 40 bees); **C)** Similar experiment but with stimulations of the antennae, the mouthparts, the front legs and the mid-hind legs (n = 37). Thermal stimulation mostly induced stronger responses than tactile controls (Mc Nemar test, ***: p<0.001). Different letters indicate significant differences between structures (Mc Nemar test, p<0.0166).

In this experiment, we aimed to determine whether controlled temperature stimulation of honeybee sensory structures can trigger a sting extension response (SER). A recent study showed that a temperature-sensitive receptor, the so-called HsTRPA, is present on several sensory structures including the antennae, the mouthparts and the legs [35]. We thus chose to study temperature sensitivity on these structures, in combination with other body parts as control. Bees were harnessed in individual holders allowing visual observation of the SER (Fig. 1A).

In a first experiment (n = 40), we evaluated the effect caused by a 1 sec stimulation with a copper probe at 65°C applied on the antennae, the mouthparts, the ventral abdomen or the dorsal abdomen (Fig. 1B). As control, an identical stimulation with an unheated probe (‘tactile control’) was applied on each structure. Stimulations were given at 10 min intervals and their order was randomized across animals. Thermal stimulations induced between 18.5% and 87.5% SER depending on the contacted structure, while tactile controls triggered less than 15% SER on all structures. Responses were significantly higher for thermal stimulation than for tactile control in the case of the antennae (Mc Nemar test, Chi^2^ = 20.0, p<0.001), the mouthparts (Chi^2^ = 33.0, p<0.001) and the ventral abdomen (Chi^2^ = 8.10, p<0.01) but not for the dorsal abdomen (Chi^2^ = 0.00, NS). Overall, the effect of thermal stimulations differed according to the contacted structure (Cochran’s Q test, Q = 44.9, p<0.001, 3 df), while no difference appeared for tactile controls (Q = 7.33, NS, 3 df). Antennal and mouthpart stimulation induced significantly higher responses than other areas (Mc Nemar test, Chi^2^>5.88, p<α_corr_ = 0.0167), but stimulations of these two organs did not differ statistically (Chi^2^ = 3.5, NS).

In a second experiment (n = 37), we reproduced the previous measures of thermal stimulation of the bees’ antennae and mouthparts and compared them with stimulations of the bees’ legs (Fig. 1C). With a different holding position, which allowed stimulating the bees’ legs with the heated copper probe, it was possible to stimulate selectively the front legs (one after the other) or the middle and hind legs (all together). The four thermal stimulations triggered from 32.4% to 94.6% SER, whereas tactile stimulations induced less than 18.9% responses. In all cases, responses induced by thermal stimuli were significantly higher than responses to tactile controls (Mc Nemar test, Chi^2^>9.09, p<0.01). Overall, the effect of thermal stimulations differed according to the contacted structure (Cochran’s Q test, Q = 40.5, p<0.001, 3 df), while no difference appeared for tactile controls (Q = 7.80, NS, 3 df). In this experiment, responses to thermal stimulation were equivalent for the antennae, the mouthparts and the front legs (McNemar test, Chi^2^<4.00, NS), while all three differed with thermal stimulation of the hind legs (Chi^2^>12.0, p<α_corr_ = 0.0167 in all cases).

These results show several structures on the bees’ body are sensitive to temperature and their stimulation triggers a defense response by the extension of the sting. Among the tested structures, the antennae, the mouthparts and the front legs were especially responsive to thermal stimulation.

### Experiment 2: Honeybees’ Sensitivity to Temperature

The previous experiment showed that stimulation of antennae, mouthparts and front legs with a high temperature (65°C) can trigger strong SER in bees. In the present experiment, we evaluated the effect of increasing temperatures on SER levels, aiming to determine the heat sensitivity of these sensory structures. Thus, temperature of the copper probe was increased from ambient temperature (∼25°C) to 75°C in steps of 10°C. Each group of bees was stimulated on the antennae, the mouthparts or the front legs with increasing temperatures, alternating with tactile controls. Intervals between stimulations were 10 min.

**Figure 2:**
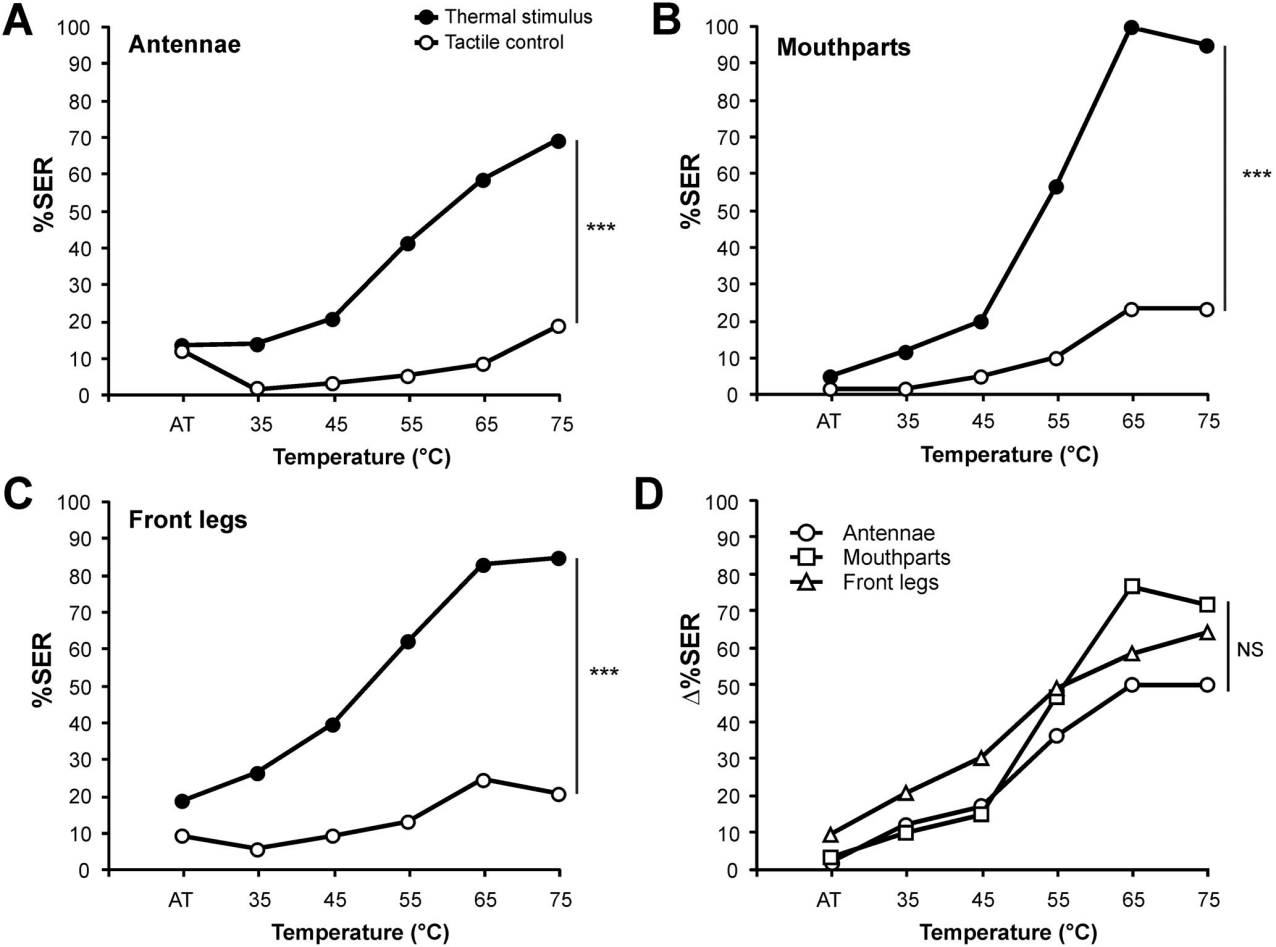
Thermal responsiveness of bees when stimulated on different structures with increasing temperatures. **A–C)** Percentage of SER to increasing temperatures (black dots, AT: ambient temperature ∼25°C, 35°C, 45°C, 55°C, 65°C, 75°C) alternating with tactile controls (white dots). Stimulations were applied on: **A)** the antennae (n = 58); **B)** the mouthparts (n = 60); **C)** the front legs (n = 53). On all three structures, bees respond differently to the thermal stimulus than to the tactile control, as a response increase is observed only with the thermal stimulus (repeated measure ANOVA, *stimulus* x *trial* effect, ***: p<0.001). **D)** Delta values (ΔSER%) resulting from the difference between the responses to the thermal and to the tactile stimuli for the three tested structures. No difference appeared in the evolution of the three curves with increasing temperature (repeated measure ANOVA, *stimulus x trial* interaction: NS).

We first focused on heat sensitivity of the antennae (Fig. 2A, n = 58). Responses increased significantly with increasing temperature, from 12.1% at ambient temperature to 62.9% at 75°C (repeated measurement ANOVA, F_5,285_ = 22.0, p<0.001). In the mean time, bees’ responses to tactile stimulation also varied during the experiment, but remained low (below 20%, F_5,285_ = 3.56, p<0.01). Accordingly, responses evolved differently along trials for thermal and tactile stimulation (*stimulus x trial* repeated measurement ANOVA, interaction: F_5,285_ = 13.2, p<0.001). Thus, thermal stimulation of the antennae induces a gradual increase in SER response with increasing temperature.

Similar observations were made when applying thermal stimulations on the mouthparts (Fig. 2B, n = 60) and on the front legs (Fig. 2C, n = 53). In both cases, SER increased with increasing temperature (repeated measurement ANOVA, mouthparts: F_5,295_ = 116.4, p<0.001: front legs: F_5,260_ = 37.6, p<0.001), reaching 100% (65°C) and 84.4% (75°C) for mouthparts and front legs respectively. Responses to the tactile control also varied throughout the experiment (mouthparts: F_5,295_ = 8.02, p<0.001: front legs: F_5,260_ = 3.84, p<0.001), increasing from 1.7–9.4% at the start of the procedure and reaching 23.3% and 20.7% respectively for mouthparts and front legs at the fifth tactile stimulation. This effect is attributable to sensitization due to the temperature stimulations. However, in both cases, responses evolved differently along trials for thermal and tactile stimulation (*stimulus x trial* interaction, mouthparts: F_5,295_ = 37.6, p<0.001; front legs: F_5,260_ = 13.9, p<0.001).

To compare thermal responsiveness of the three structures independently of sensitization, we computed for each bee and at each trial a delta value (Δ%SER), resulting from the difference between its response to the thermal and to the tactile stimulus. Figure 2D shows the delta values for the antennae, the mouthparts and the front legs. A global analysis of these curves indicated a significant difference among structures (*structure x trial* repeated measure ANOVA, *structure* effect, F_2,168_ = 3.37, p<0.05). This effect was probably due to higher delta values for stimulation of the front legs compared that of the antennae, although the posthoc comparison was only near-significant due to multiple comparison correction (Tukey HSD test, p = 0.047>α_corr = _0.025). However, the evolution of responses with increasing temperature was similar as the *stimulus x trial* interaction was not significant (F_10,840_ = 1.73, NS).

These results show that thermal stimulation of the antennae, mouthparts or front legs induces a gradual increase in SER response with increasing temperature. This experiment also indicates that 65°C corresponds to an optimum across structures for triggering SER in most individuals. It may thus qualify as an efficient US for aversive conditioning.

### Experiment 3: Thermal Aversive Conditioning

Given that a thermal stimulation of the antennae, mouthparts or front legs triggers a SER, we addressed the possible function of such thermal stimulus as an US in aversive SER conditioning. We thus performed a differential conditioning procedure in which an odorant was associated with a stimulation with the copper probe at 65°C (CS+) and another odorant was presented without reinforcement (CS−). Each bee thus received 8 CS+ and 8 CS− trials in a pseudo-randomized order. Three groups of bees were thus conditioned, with the US applied on the antennae, the mouthparts, or the front legs. In each group, half of the individuals received the reinforcement when the odorant 2-octanone was presented and no reinforcement when nonanal was presented, while the reversed combination was used for the other half. The inter-trial interval was 10 min.

**Figure 3:**
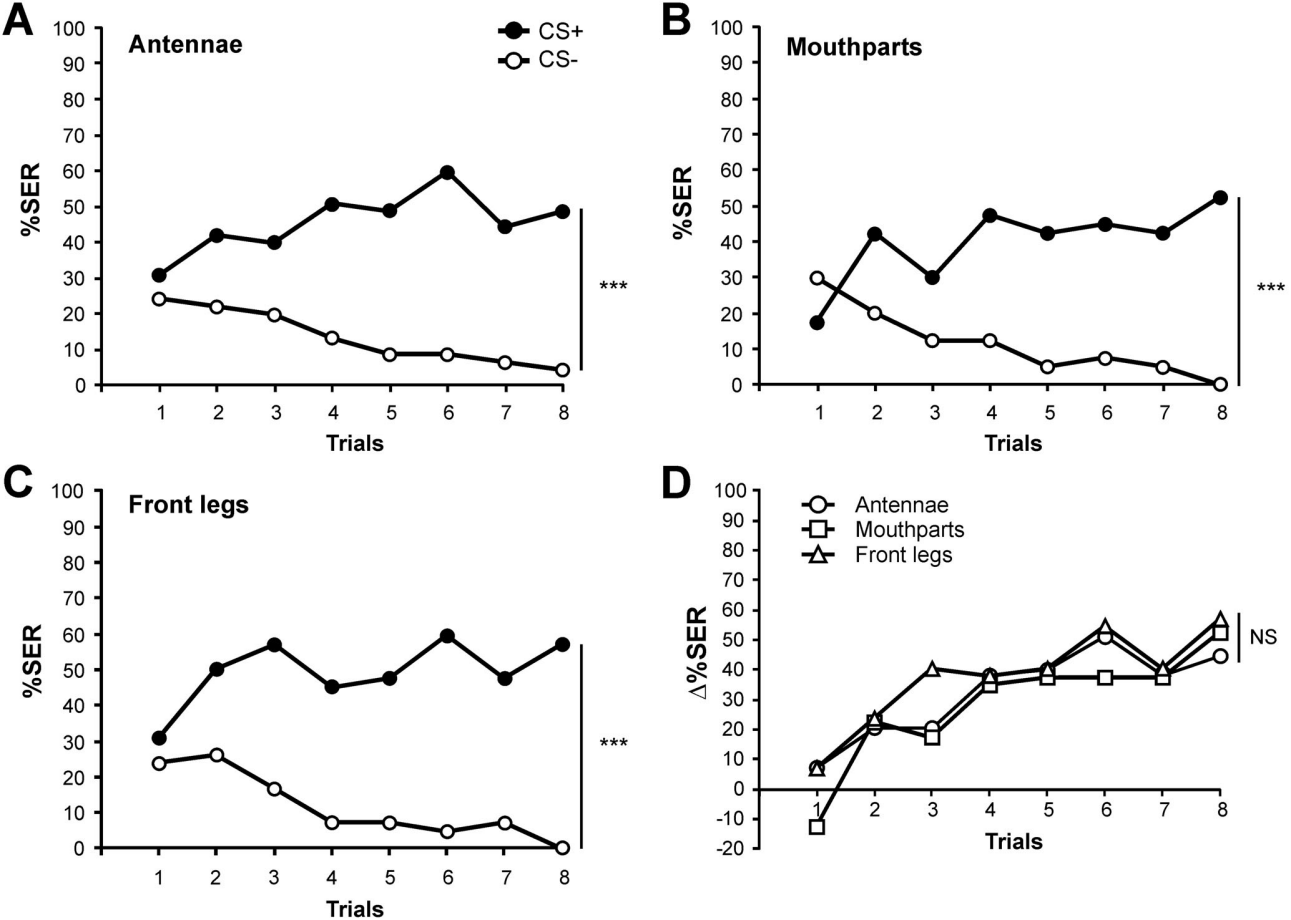
Thermal aversive conditioning with the US applied on different structures. **A–C)** Percentage of SER to the reinforced odorant (CS+, black dots) and to the non-reinforced odorant (CS−, white dots) along conditioning trials. The thermal unconditioned stimulus (65°C) was applied on: **A)** the antennae (n = 45); **B)** the mouthparts (n = 40); **C)** the front legs (n = 42). Bees learn to respond to the CS+ and not to the CS− when the thermal stimulus is provided on any of the three structures (repeated measurement ANOVA, *stimulus* x *trial* interaction: ***: p<0.001). **D)** Delta values (Δ%SER) resulting from the difference between the responses to the CS+ and to the CS− for the US applied on the three tested structures. No difference appeared in the evolution of the three curves along conditioning trials (repeated measure ANOVA, *stimulus x trial* interaction: NS).

For all three structures, the two subgroups did not show any response difference along trials (ANOVA for repeated measurement, antennae: F_1,43_ = 0.03, NS; mouthparts: F_1,38_ = 0.08, NS; front legs: F_1,40_ = 0.05, NS) and, hence, were pooled for the analysis. Figure 3A presents the results for the group receiving the US on the antennae (n = 45). Along the trials, bees’ responses to the reinforced (CS+) and to the non-reinforced odorant (CS−) developed differently (ANOVA for repeated measurement, *stimulus* x *trial* interaction: F_7,308_ = 5.07, p<0.001). Responses to the CS+ increased (ANOVA for repeated measurement: F_7,308_ = 2.44, p<0.05), while responses to CS− decreased (ANOVA for repeated measurement: F_7,308_ = 3.00, p<0.01). Thus bees are able to associate an odorant with a thermal US to the antennae. Similarly, we examined aversive conditioning with the thermal US applied to the mouthparts (Fig. 3B, n = 40) and to the front legs (Fig. 3C, n = 42). In both cases, responses to the CS+ and to the CS− developed differently along trials (*stimulus* x *trial* interaction, mouthparts: F_7,273_ = 7.92, p<0.001; front legs: F_7,287_ = 4.93, p<0.001). Responses to the CS+ increased (mouthparts: F_7,273_ = 3.47, p<0.01; front legs: F_7,287_ = 2,27, p<0.05) whereas responses to the CS− decreased significantly (mouthparts: F_7,273_ = 4.51, p<0.001; front legs: F_7,287_ = 4.36, p<0.001). Thus, bees learned to respond to the CS+ and to not respond to the CS−.

To compare the aversive learning performances between the three groups which received the thermal US on different structures, we computed for each bee and at each trial a delta value (Δ%SER), resulting from the difference between its response to the CS+ and to CS−. Figure 3D shows the delta values for groups reinforced aversively on the antennae, the mouthparts and the front legs. A global analysis of these curves did not show any significant difference among structures (*structure x trial* repeated measure ANOVA, *structure* effect, F_2,124_ = 1.16, NS). In addition, the three groups learned as quickly to differentiate the odorants as the *stimulus x trial* interaction was also not significant (F_14,868_ = 0.74, NS).

We thus conclude that thermal reinforcement can be used as US in SER aversive conditioning regardless of whether the temperature stimulation is applied on the antennae, the mouthparts or the front legs. Thermal stimulations of the three structures are equally efficient as aversive US.

### Experiment 4: Genotypic Influence on Thermal Responsiveness and Aversive Learning

The previous experiments showed that the percentage of individuals showing a SER to a thermal stimulation increases gradually with the temperature of the stimulation. This observation suggests individual differences in bees’ sensitivity to temperature. In addition, although bees as a group learned to associate odorants with a thermal US, their individual performances varied with some bees learning quickly and efficiently and other bees not learning the association at all. Previous work suggested that at the individual level, bees’ aversive learning performances depend on their sensitivity to an electric shock US [30]. In the present experiment we aimed to confirm this finding with a thermal US. In addition, we aimed to understand the possible genotypic origin of such inter-individual differences in thermal sensitivity and/or aversive learning performance.

In this experiment, we used only 13–14 day-old bees, to avoid any influence of bees’ age. Bees were subjected to a thermal responsiveness experiment (as in Experiment 2) followed by an aversive olfactory conditioning protocol (as in Experiment 3). Thermal stimulations were applied to the mouthparts as this showed the strongest SER rate in previous experiments. For assessing the putative genetic dependency of thermal sensitivity and aversive learning performances, all individuals were genotyped based on a set of 14 microsatellite markers, allowing to determine their patriline of origin.

**Figure 4:**
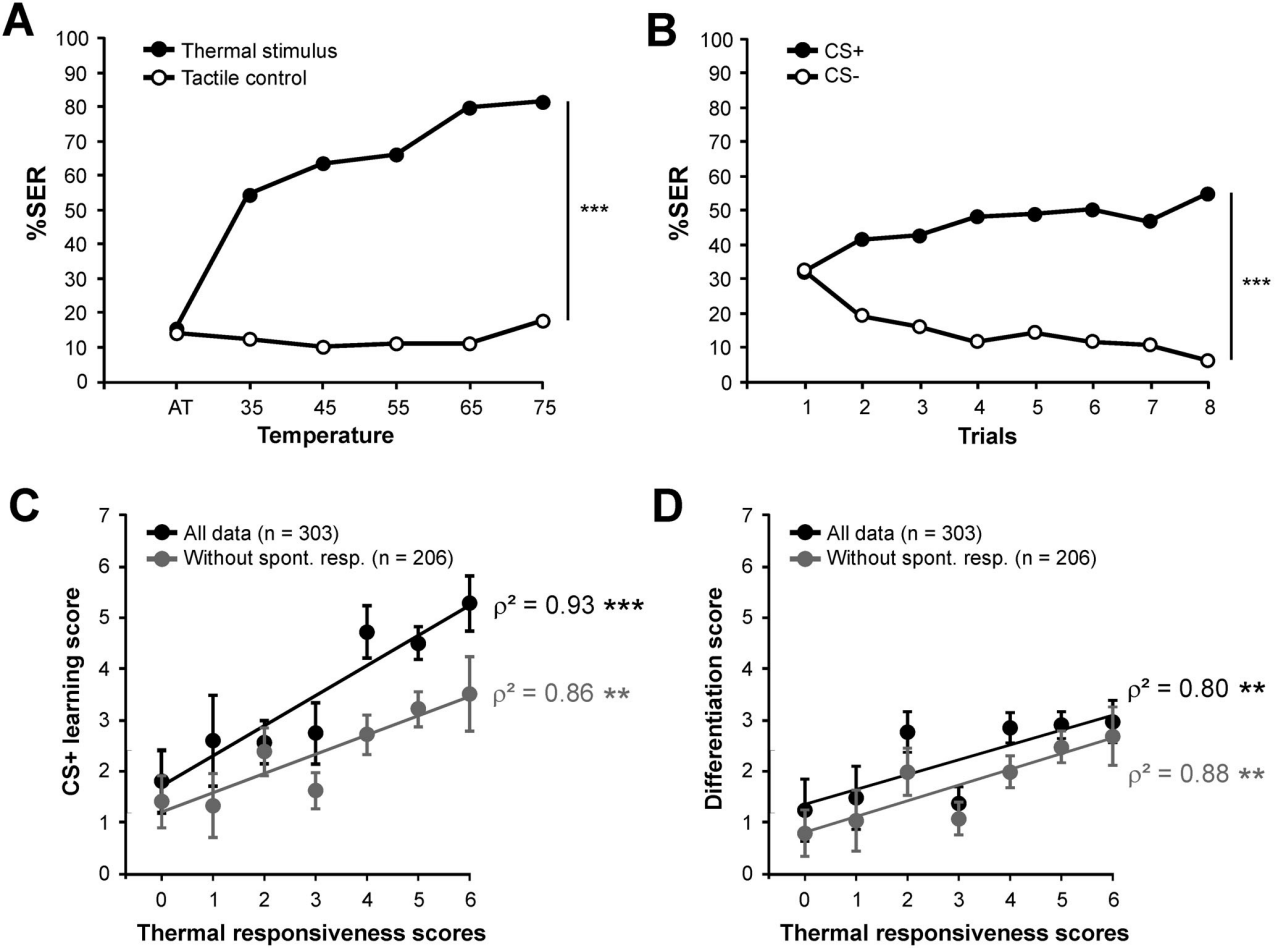
Measure of thermal responsiveness and aversive learning performance on the same bees. **A)** Thermal responsiveness curve with the temperature stimulus provided on the mouthparts (n = 303). Percentage of SER with increasing temperatures (black dots) or with tactile control (white dots). The curves for thermal and tactile stimuli develop differently (repeated measure ANOVA, *stimulus* x *trial* effect, ***: p<0.001). **B)** Aversive learning performances with thermal reinforcement on the mouthparts (n = 303). Percentages of SER to the CS+ (black dots) and to the CS− (white dots). Bees learned to respond to the CS+ and not to the CS− (repeated measure ANOVA, *stimulus* x *trial* effect, ***: p<0.001). **C)** Relationship between thermal responsiveness and aversive learning performance. The graph shows average response to the CS+ (± SEM) for bees with different thermal responsiveness scores (n = 17–81 per score). A significant linear relationship between the two variables is found, both using all data (black dots) or only those from bees that did not respond spontaneously to the CS+ (grey dots) (Spearman correlation, ***: p<0.001; **: p<0.01; 8 df). **D)** Relationship between thermal responsiveness and differentiation performance in the differential conditioning. The graph shows average delta values (responses to the CS+ minus responses to the CS−) ± SEM for bees with different thermal responsiveness scores (n per score as in C). A significant linear relationship between the two variables is found, both using all data (black dots or only those from bees that did not respond spontaneously to the CS+ (grey dots) (Spearman correlation, **: p<0.01; 8 df).

Thermal responsiveness (Fig. 4A, n = 303) and aversive conditioning (Fig. 4B, n = 303) yielded similar results as in the previous experiments, except that bees in this experiment appeared generally more sensitive to temperature (i.e; they responded at lower temperature) than in Experiment 2. This is probably due to the fact that the two experiments were performed at different periods of the year (Exp. 2: February–March; Exp. 4: May–June). In any case, in the thermal responsiveness experiment (Fig. 4A), responses increased with increasing temperature (F_5,1510_ = 126.9, p<0.001) while response to tactile stimulations remained below 18%, but showed significant variations along the procedure (F_5,1510_ = 2.72, p<0.05). Responses to thermal and tactile stimuli developed differently along the procedure (*stimulus x trial* repeated-measurement ANOVA, interaction: F_5,1510_ = 82.0, p<0.001). In the differential conditioning protocol (Fig. 4B), bees learned to respond to the CS+ (F_7,2114_ = 12.2, p<0.001) and to not respond to the CS− (F_7,2114_ = 23.9, p<0.001) so that responses to both stimuli developed differently along trials (*stimulus* x *trial* repeated measurement ANOVA, interaction: F_7,2114_ = 36.7, p<0.001).

Based on these results, we calculated for each bee its *thermal responsiveness score* as the number of responses to the thermal stimuli (from 0 to 6). Thus, a bee with a high score is highly sensitive to temperature, as it would start responding already at rather low temperatures. Likewise, we calculated for each bee its *aversive learning score,* as the number of responses to the CS+ (from 0 to 8). A bee with a high score would be a good aversive learner, which learned quickly to respond to the reinforced odorant. We then asked whether bees’ learning performance can be predicted based on their responsiveness to the thermal US. Figure 4C (black dots) presents the average aversive learning score for bees showing a particular heat responsiveness score. A clear linear relationship can be observed, as the more thermally responsive bees (i.e. more sensitive to temperature) show higher aversive learning scores. Accordingly, aversive learning scores differ among thermal responsiveness score categories (one-way ANOVA, F_6,181_ = 5.34, p<0.001) and the linear relationship between both variables is highly significant (Spearman correlation, ρ^2^ = 0.93, p<0.001, 8 df).

As at the start of conditioning, about one third of the bees responded spontaneously to the CS+ (see Fig. 4B), the previous measure of the *aversive learning score* over all tested individuals could be considered potentially spurious, since individuals that are highly sensitive to the US may also be sensitive to other stimulations and respond spontaneously with a SER to odorants. We thus performed the previous comparison taking into account only bees which did not respond spontaneously to the CS+ (n = 206, score 0 to 7). As Fig. 4C (grey dots) shows, without spontaneous responders, the linear relationship between thermal responsiveness and aversive learning is almost fully conserved (Spearman correlation, ρ^2^ = 0.86, p<0.01, 8 df). Thus, spontaneous responses cannot explain the strong relationship we observed.

As a further verification, we also calculated for each bee a *differentiation score*, as the difference between the number of responses to the CS+ and to the CS− over the course of the experiment. A value of 0 would mean that the animal does not learn to respond to the CS+ and not to the CS−, while increasing positive values indicate increasing levels of differentiation between CS+ and CS−. It is therefore a purely associative measure of aversive learning success, which contains its own control for non-associative responses. Again, there was a highly significant linear relationship between *thermal responsiveness* and the *differentiation score*, both for all bees (black dots, ρ^2^ = 0.80, p<0.01, 8 df) and for non-spontaneous responders (grey dots, ρ^2^ = 0.88, p<0.01, 8 df). We thus conclude that bees’ responsiveness to the thermal US determines their aversive learning performance with this US.

**Figure 5:**
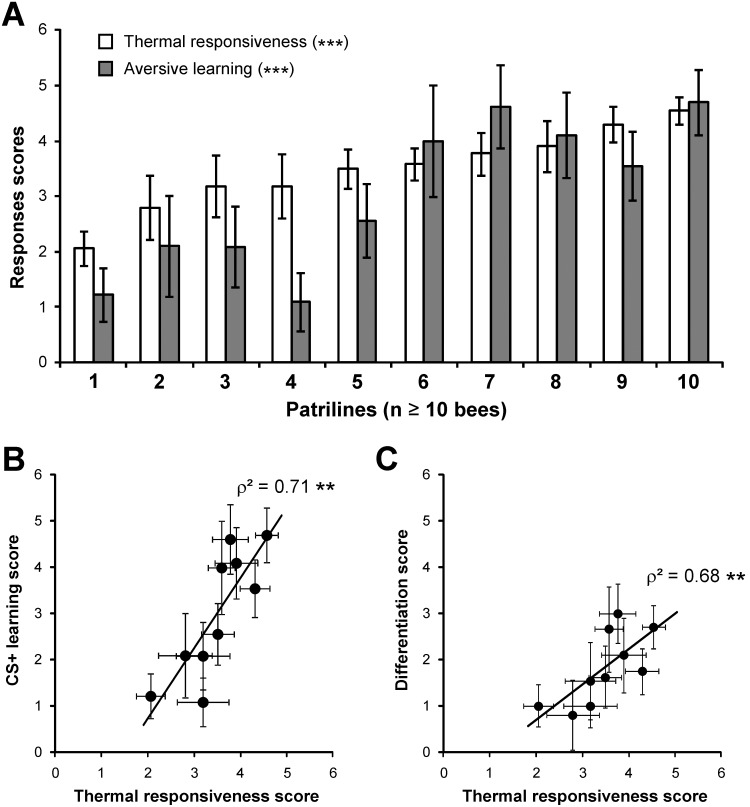
Genotypic influence on thermal responsiveness and aversive learning (patriline effect). **A)** Thermal responsiveness (white bar, average ± SEM) and aversive learning scores (grey bar, average ± SEM) for the 10 patrilines with the most samples (n = 10–27 bees per patriline). Patrilines are ranked according to increasing thermal responsiveness scores. Significant differences among patrilines are observed for both scores (one way ANOVA, p<0.001). **B)** A strong correlation appears between thermal responsiveness and aversive learning performances at the patriline level (Spearman correlation, **: p<0.01, 8df). **C)** Likewise, a significant correlation appears at the patriline level between the differentiation score (difference between responses to the CS+ and to the CS−) and the thermal responsiveness score (Spearman correlation, **: p<0.01, 8 df).

We next asked what may drive the observed inter-individual differences in thermal responsiveness and learning. Using a microsatellite analysis, which enabled us to determine the patriline origin of each bee, we assessed the impact of genotype on the thermal responsiveness/aversive learning relationship. The 303 individuals tested in this experiment belonged to 22 different patrilines (i.e. were sired by one of 22 drones which mated with the queen). The numbers of bees within each patriline ranged from 1 to 27 individuals. For assessing patriline performance scores accurately, we only used data from the 10 patrilines which contained more than 10 individual bees. Figure 5A presents average thermal responsiveness and aversive learning scores for these 10 patrilines. Among these patrilines, significant differences were observed in both thermal responsiveness (one way ANOVA, F_9,138_ = 4.37, p<0.001) and aversive learning scores (F_9,138_ = 3.59, p<0.001). Generally, bees from patrilines with a high (resp. low) responsiveness to thermal stimuli also had a high (resp. low) learning score. Accordingly, a strong correlation was observed at the patriline level (Fig. 5B, ρ^2^ = 0.71, p<0.01, 8 df). Likewise, when using patrilines’ *differentiation score*, measuring the differentiation between CS+ and CS−, a clear and significant correlation was observed (Fig. 5B, ρ^2^ = 0.68, p<0.01, 8 df). Thus aversive learning performance and sensitivity to the thermal US are under clear genotypic influence and are strongly linked. Within this general trend, however, some deviations could be observed. For instance, while patrilines 3, 4, 5 and 6 display similar thermal responsiveness scores, their aversive learning scores are different. Therefore, in addition to thermal responsiveness, aversive learning performance is also under the influence of other – untested – genetic traits.

## Discussion

This study first shows that a thermal stimulus applied on different parts of the bee's body can trigger a sting extension response (SER). Most responses were observed when the thermal stimulus was applied on the mouthparts, the antennae or the front legs, suggesting that these structures are the most sensitive to temperature. We then established the use of such thermal stimuli as US in aversive olfactory conditioning of the SER. In a differential conditioning procedure, bees responded more to the CS+ than to the CS− when the thermal US was given to the antennae, the mouthparts or the front legs. Thus thermal stimulation of all three structures can serve as aversive US in SER conditioning. We found a clear correlation between bees’ responsiveness to thermal stimuli and aversive learning performance, both at the individual and at the patriline level. Different patrilines within the hive displayed different sensitivities to the US, and accordingly different aversive learning performances. These results establish for the first time a strong genotypic influence for aversive conditioning in honeybees.

### Temperature Detection in the Honey Bee

The first important observation of this study is that a thermal stimulus applied on the bee's body triggers SER, which can be interpreted as a defense reaction of the bee towards potentially noxious stimulations. In addition to the advantage of using this stimulus as US in aversive conditioning (see below), this observation provides an interesting means of studying heat sensitivity in honeybees. Thus, in the first part of this work, we measured bees’ responses when the thermal stimulus was applied on different sensory structures. Five structures showed significant responses to temperature compared to tactile controls. Among those, three crucial sensory organs of bees (antennae, mouthparts and front legs) induced the strongest SER levels. The antennae are prominent sensory organs (mostly olfactory, tactile and gustatory) in which thermal detection was already known, as they harbor specific thermo-sensitive sensilla (coelocapitular sensilla, [36]). Furthermore, at the behavioral level, the antennae are crucial for the avoidance of high temperatures by freely-walking bees [35]. However, thermal sensitivity at the level of the mouthparts and the front legs had not been precisely described before, although heat detection by these organs seems coherent for maintaining the insect’s integrity. One can hypothesize that thermal sensitivity at the level of the mouthparts could be adaptive for avoiding food sources at temperatures that could cause internal injury. Thermal sensitivity at the level of the bees’ legs could be crucial to avoid landing on hot surfaces during summer months. These ideas are consistent with the recent discovery of the first honeybee thermal receptor within these three sensory organs [35]. In contrast to these structures, we did not observe any significant effect of thermal stimulation on the dorsal abdomen. Possibly, thermo-sensitive receptors are not expressed in this region or thermo-sensitive cells are not linked to motor output leading to SER. Apart from this last case, thermal sensitivity seems however broadly represented on the honeybees’ body and SER may allow precisely mapping this sensitivity.

### Thermal Stimulation as US in Olfactory Aversive SER Conditioning

We show that a thermal stimulus applied to the antennae, the mouthparts or the front legs can act as a US in aversive SER conditioning. Temperature represents an interesting alternative to the electric shock for studying aversive learning, as it is a more natural stimulus for bees and it can be applied more locally on the bees’ body. Moreover, prior identification of thermo-sensitive sensilla [36]–[37] and receptors [35] could be advantageous for building a neural model of aversive conditioning in bees, based on identified sensory structures and neuronal pathways [8]. In theory, associative learning is possible because at one or several locations in the brain, the CS and US pathways converge and neural plasticity takes place at these locations. The olfactory (CS) pathway has been well described in honeybees [6]–[7], [38]: olfactory receptor neurons located on each antenna project to the antennal lobes where primary olfactory processing takes place. From there, projection neurons convey processed information to higher-order brain centers, the mushroom bodies and the lateral horn. For aversive learning, the US pathway is mostly unknown, but our results may provide some new clues. In the case of conditioning with an antennal temperature US (Fig. 3A), thermo-sensory neurons from coelocapitular sensilla on the antenna are thought to project to the antennal lobe [36], [39]. In another Hymenoptera, the ant *Atta vollenweideri*, an optical imaging study showed that a temperature change in the stimulation airflow induced clear patterns of activity in several glomeruli of the antennal lobe [40]. A first direct convergence between olfactory (CS) and thermal (US) pathways may thus be found in this structure. Successful aversive learning was also observed with a thermal US on the mouthparts (Fig. 3B) and the front legs (Fig. 3C). Data in other insects suggest that putative thermo-sensitive neurons on these structures would first project to the respective ganglia of the ventral nerve cord, respectively to the subesophageal and prothoracic ganglia [41]. From there, information could be conveyed by interneurons towards the brain, possibly to a thermal integration center, as suggested by several observations. In *Drosophila*, thermal neurons from the arista project to the proximal antennal protocerebrum, a region between the antennal lobe and the sub-esophageal ganglion [42]. This structure contains at least two subregions, one responding to cold, and another to warmth. In the bees *Apis cerana*, immediate early gene expression mapping showed that exposure to a high temperature (46°C) induces neural activity in a region of the protocerebrum located between the dorsal and the optic lobe [43]. Neurons from such a putative thermo-sensory center would then activate aversive reinforcement circuits, which would converge with the olfactory pathway and induce learning-associated plasticity. Dopaminergic neurons are thought to mediate aversive reinforcement in the bee brain because pharmacological blockade of dopamine receptors disrupts aversive learning [31]. Dopamine neurotransmission is also necessary for aversive learning in other insects (*Drosophila*, [44]–[45]; crickets, [46]). The bee brain contains a complex arrangement of dopamine-immunoreactive neurons [47]–[48]. Among them, three clusters contain processes that project to the mushroom body calyces and lobes (especially the α-lobe), and may thus provide aversive reinforcement information to the olfactory pathway [8]. Neuroanatomical and neurophysiological work (electrophysiology, optical imaging) will be needed to confirm these putative circuits.

### Relationship between US Sensitivity and Aversive Learning Performance

Associative learning performance usually depends on an animal’s sensitivity to both the CS and the US. In honeybees, previous work on appetitive conditioning has established the strong influence of sucrose (US) sensitivity on learning performances. Bees with a low response threshold, i.e. which are highly sensitive to sucrose, learn better than bees with a higher threshold, as they give a higher subjective value to the US [26]–[27]. Likewise, it was recently demonstrated that a high electric shock sensitivity leads to better aversive learning performances [30]. We confirm and extend this relationship. In the former demonstration [30], bees were divided into two groups depending on their sensitivity to the electric shock (low *vs* high) precluding a true correlative analysis. By dividing bees in 7 thermal responsiveness score groups, we show a clear linear correlation between thermal responsiveness and aversive learning scores, suggesting that the more sensitive a bee is to temperature, the better it can learn to associate an odor with this US. The potentially confounding effect of high spontaneous responses observed in SER conditioning was excluded, as the correlation remained when removing spontaneous responders (Fig. 4C) or when focusing on the response difference between CS+ and CS− (*differentiation score*, Fig. 4D).

### Genetic Influence on Thermal Sensitivity and Aversive Learning

In our study, the relationship between aversive conditioning and US sensitivity was considered with a special emphasis on its genetic determinism. We show here that bees’ genotype influences their thermal responsiveness and hence affects their aversive learning performances with a thermal US. Previous work had shown that different patrilines react differently to a fixed-intensity aversive stimulus (electric shock; [49]). However, no study had evaluated the differential sensitivity of bees from different patrilines to a series of aversive stimuli of increasing intensity, nor had aversive learning performances been evaluated as a function of patriline origin. Although we do not know the influence of maternal genotype on aversive responsiveness and learning, the strong paternal effect we have found is coherent with previous crosses performed between European and Africanized honeybees which showed that drone-inherited genes more strongly determine defensive behavior at the colony level than the queen’s genes [50]. Concerning *appetitive* behavior, the genetic dependency of sucrose responsiveness is well known. For instance, two strains of bees selected for pollen hoarding (amount of pollen stored in the colonies) show a different sucrose responsiveness (PER), and accordingly different tactile and olfactory learning performances with a sucrose US [24], [28]. In addition, it was recently shown that sucrose responsiveness is different among patrilines from the same hive [51]. In the same logic, we found a clear genotypic influence on thermal responsiveness. As aversive and appetitive learning are thought to correspond to two mostly independent modules of honeybees’ behavior (foraging and defense respectively, [30]), an important question for future work will be to understand the relative dependency of genes involved in each learning form. At this stage, we know that sucrose responsiveness and electric shock responsiveness tested in the same bees are not correlated [30]. It will be important next to extend this finding to thermal sensitivity and to ask how the aversive and appetitive learning performances of bees from different patrilines are related.

Genetic differences in thermal sensitivity may arise at multiple levels. First, peripheral thermal receptors may be differentially expressed among patrilines. For instance, if we assume that the TRP channel HsTrpA previously identified in bees is responsible for thermal detection in our protocol, it could exist in different allelic forms in different patrilines or its expression may be differently regulated. Similarly, in the central nervous system, alleles or expression levels of crucial effectors for heat sensitivity may differ. A possible example would be bees’ ortholog of the voltage-gated calcium channel subunit *straight-jacket* of *Drosophila* or *CACNA2D3* (α2δ3) of mice, which is implicated in heat pain sensitivity in both animals [52]. Additionally, dopamine is considered as the neurotransmitter conveying aversive reinforcement information in the insect brain [31], [44]–[45], [53]. Different patrilines may produce different levels of this neurotransmitter and/or may express its receptors (AmDop 1, 2 and 3) differentially. Lastly, genetic differences among patrilines may induce some epigenetic modifications known to be part of the task allocation process in a bee hive [54]–[55]. DNA methylation can influence some aspects of learning and memory processes in bees [56]–[57]. Enzymes responsible for DNA methylation may be more or less active in different patrilines. By altering chromatin structure or regulating transcriptional machinery, differentially methylated regions (DMRs) could potentially influence the expression of genes involved in aversive learning or thermal sensitivity.

Although thermal sensitivity strongly influenced aversive learning performances, it did not explain all the learning differences observed among patrilines. For instance, some patrilines showed similar thermal sensitivity but different learning performance levels (see Fig. 5A). In this case, genetic differences may appear due to differences in bees’ sensitivity to the odor CS, for instance through differential expression of olfactory receptors (ORs) or through differential wiring at multiple levels within olfactory circuits. However, the observed heterogeneity among patrilines with equal thermal sensitivity may reveal ‘real’ differences in learning ability, which may relate to different alleles or expression levels of CS-US association enzymes, like adenylate cyclases (AC) or other molecular actors of acquisition or memory formation [58]–[59]. For this reason, it is important to compare the influence of genetics on these different aspects: sensitivity to the CS, sensitivity to the US, association machinery. The present study shows a strong influence of US sensitivity but suggests a non-negligible role of the other determinants.

### General Outlook

How may genetic variability in learning and memory abilities influence colony fitness and survival? It has been proposed that a higher genetic variability (for instance, more numerous patrilines) within a social insect colony may allow more flexibility and a higher capacity to cope with changes in environmental conditions, by providing different types of genetically-specialized individuals especially efficient for carrying out particular tasks (cleaning, nursing, foraging, defense, etc.) [10]. For instance, a higher number of patrilines is beneficial for thermal regulation, as bees from different patrilines engage in fanning activity at different deviations from the optimal temperature, thereby providing a gradual and more efficient response to outside temperature changes [13]. In a social insect colony, the different patrilines are not equally involved in the different tasks [60]–[62] and workers performing different tasks show different associative learning abilities (appetitive modality: [63]–[64]; aversive modality: [30]). It will now be important to compare appetitive and aversive learning abilities in different patrilines and to relate these differences with the tasks these individuals actually carry out in the hive. Such experiments shall help us understand to which extent task allocation is based on a genetic determinism of aversive or appetitive learning capacities.

## Materials and Methods

### Animals

Experiments were performed on honeybees (*Apis mellifera L.*) captured from outdoor hives located at the CNRS campus of Gif-sur-Yvette, between January and November 2011.

### Experiment 1: Effect of Temperature on the Sting Extension Response

We first aimed to determine whether thermal stimulation of several structures on the bees’ body could trigger a SER. Bees were taken from the hive in the morning and chilled on ice until they stopped moving. Then, they were harnessed into individual holders, similar to those usually used for PER conditioning [17], [65]. The position of the honeybee in the holder was however different from that used in PER conditioning. The bee was placed with its back towards the front of the tube, with a piece of tape placed below the head to the front and at the thorax level (Fig. 1A). Thus, the abdomen could move freely and bees’ SER could be observed throughout the experiment. Thermal stimulation was provided by means of a pointed copper cylinder (widest diameter: 6 mm; length: 13 mm), mounted onto the end of a minute soldering iron running at low voltage (HQ-Power, PS1503S). Temperature at the end of the cylinder was controlled, at the beginning and at the end of each experiment, using a contact thermometer (Voltcraft, Dot-150). Thermal stimulations were applied during 1 s on six different areas of the bees’ body: the antennae (both flagella simultaneously), the mouthparts (the different articles were stimulated simultaneously, indiscriminately; the proboscis was never extended), the front legs (one after the other, as they were fixated too widely apart for stimulating both simultaneously), the mid- and hind legs (simultaneously), the ventral abdomen (sternites of segments #3 to5), and the dorsal abdomen (tergites of segments 3 to 5).

To avoid any fatigue of the bees, only 4 structures were tested per bee. In one experiment, bees were stimulated on the antennae, the mouthparts, the ventral and the dorsal abdomen. In a second experiment, a new set of bees was stimulated on the antennae, the mouthparts (replications of the former), the front legs and the mid/hind legs. In this last experiment, the front legs were fixated with thin tape strips on each side of the harnessing tube to facilitate stimulation with the copper probes.

We applied tactile controls on the same structures, to insure that sting extension was really a consequence of thermal stimulation. Tactile stimulations were performed with a duplicate copper probe which remained at ambient temperature. For each bee, the order of stimulation of the different structures, as well as whether each stimulation was performed with the heated or with the control probe, were determined randomly prior to starting the experiment. Stimulations were performed at 10 min intervals. In this experiment, two groups of 20 bees were tested each day.

### Experiment 2: Honeybees’ Sensitivity to Temperature

Honeybees were collected the day before the experiment, and were kept in a plexiglass box containing honey and water *ad libitum*. The day after, they were immobilized on ice and then placed in holders as described above (first harnessing position). Two groups of twelve honeybees were prepared each day. Once mounted, bees were placed in a moist and dark container for two hours to accommodate to the holders. Bees were then stimulated with a succession of six heated stimulations of increasing temperature (from ambient temperature ∼25°C to 75°C), in steps of 10°C. Thermal stimulations alternated with tactile controls, provided as above with an identical unheated probe, with 10 min intervals between any two stimulations.

### Experiment 3: Thermal Aversive Conditioning

Bees were collected from the hive entrance in the morning. They were chilled on ice and placed in individual holders. They were then fed with 3 µL sucrose solution (50% w/w) and were placed in a moist and dark container for two hours as above. A group of 16 bees was used every day. Then, bees were subjected to a differential aversive conditioning procedure, in which one odorant (the CS+) was associated with a thermal reinforcement (the US), while another odorant was presented without reinforcement (the CS−). The chosen odors were 2-octanone and nonanal (Sigma Aldrich, Deisenhofen, Germany). Five microliters of pure odorants were applied onto a 1 cm^2^ piece of filter paper which was transferred into a 20 ml syringe (Terumo) allowing odorant delivery to the antennae.

Half of the honeybees received thermal reinforcement when 2-octanone (odor A) was presented and no reinforcement when nonanal (odor B) was presented, while the reversed contingency was used for the other half. Both groups were conditioned along 16 trials (8 reinforced and 8 non-reinforced) in which odorants were presented in a pseudo-random sequence (e.g. ABBABAAB) starting with odorant A or B in a balanced way. The inter-trial interval (ITI) was always 10 min. Each conditioning trial lasted 36 s. The bee was placed in the stimulation site in front of the air extractor, and left for 18 s before being exposed to the odorant paired with the US. Each odorant (CS+ or CS−) was delivered manually for 4 s. The thermal stimulus started 3 s after odorant onset and finished with the odorant (1 s temperature stimulation). The bee was then left in the setup for 14 s and was then removed. The temperature of 65°C was chosen for the US because this stimulation induced a high rate of SER in the previous experiments. In this experiment, thermal reinforcement was provided on the antennae, the mouthparts or the front legs, depending on the experimental group. One group of 16 bees was tested daily.

### Experiment 4: Genotypic Influence on Thermal Responsiveness and Aversive Learning

Age-controlled honey bees (13–14 days old) were used in this experiment to avoid any impact of age on bees’ behavior [24]. Every second day, a comb with enough capped brood was placed into an incubator (34°C) during one night. The day after, newly emerged bees were painted with a two-color code (Posca, France) and then placed back into the hive. Thirteen days later, the bees were taken from the hive and used in the behavioral experiments. At this age, honey bees usually start to perform tasks outside the hive such as guarding or foraging [66].

#### Thermal responsiveness and aversive learning

To compare heat responsiveness and aversive learning performances at the individual level, both experiments were performed on the same honeybees, one after the other [30]. On the first day, bees were subjected to the thermal responsiveness protocol (as above), and on the second day they followed an aversive learning procedure (as above, with 1-hexanol and 1-nonanol as odorants). The interval between the two experiments was 24 h. During this time, bees were kept in a dark wet box. As bees’ performances in Experiment 3 were high when the thermal US was provided on the mouthparts, this option was chosen in the present experiment. After the behavioral study, bees were placed individually in numbered Eppendorf tubes filled with 90% ethanol for genotyping.

#### Determination of patriline origin

To characterize the patriline origin of each tested bee, we used a microsatellites locus analysis, using 14 well-characterized loci. DNA was extracted using the 10% Chelex method [67], adapted for squashed bee head tissues [68]. Microsatellites amplifications were performed using 3 different multiplexes, which allowed analyzing several loci simultaneously. Multiplex 1 was composed of loci B124, A88, A28, A24, Ap55 and A66. Multiplex 2 was composed of loci A113, A7, Ap43 and Ap81. Multiplex 3 analyzed loci Ap33, A43, A8, Ap36. PCR conditions followed previous studies [69]–[70]. DNA fragments were identified using an ABI 3130 Genetic Analyzer and the Genscan analysis software (version 3.7.1). Allelic sizes were labeled using Genemapper 4.1. Allele nomenclature was standardized using reference samples [71]–[73]. Once the multilocus genotype of each worker bee was determined, queen genotype was deduced, looking for homozygous genotypes for each locus in the worker data set (queen progeny). The multilocus genotype of the queen was verified, using the Colony 1.2 program [74]. The program analyzes haplo-diploid systems based on the expression of codominant genetic markers, such as DNA microsatellites. It calculates the probabilities of all possible queen genotypes, based on the observed allele frequencies in the population. Paternal alleles for each worker were then characterized after subtracting the queen’s allele from each worker’s genotype. Workers were considered as belonging to the same patriline when the same alleles were shared over all (14) analyzed loci.

### Statistical Analysis

All recorded data were dichotomous, with a sting extension being recorded as 1 and a non-extension as 0. In the conditioning experiments with the thermal US on different body parts (Experiment 3), bees which did not respond three times to the US (out of 8 CS+ trials) were excluded from the analysis, as they were considered as not aversively motivated enough. They represented less than 15% of all conditioned bees. When comparing the responses of the same bees to the thermal or tactile stimulation of different structures (Experiment 1), Cochran’s Q test was used, followed by pairwise comparisons using a Mc Nemar test. To analyze thermal sensitivity curves (Experiment 2 and 4) or differential conditioning curves (Experiment 3 and 4), we used repeated measure ANOVAs with stimulus (either thermal vs tactile, or CS+ vs CS−) and trial as factors. To evaluate individual sensitivity or learning curves, one-factor repeated measure ANOVAs were used. Monte Carlo studies have shown that it is permissible to use ANOVA on dichotomous data only under controlled conditions, which are met in these experiments (highly similar frequencies and at least 40 degrees of freedom of the error term [75]).

A correlative approach was chosen to analyze relationships between thermal responsiveness and aversive learning performances at the individual and at the patriline levels (Experiment 4). We calculated for each bee its *thermal responsiveness score* (from 0 to 6) by counting the number of times it responded to the thermal stimulus presented at increasing temperatures. Higher scores indicate bees that started to respond at lower temperatures, and are thus more sensitive to temperature. In the same manner, we calculated two learning performance scores. For the *aversive learning score*, we counted the number of times bees responded to the reinforced odorant (CS+). A higher score indicated a good learner, which quickly associated the CS+ with reinforcement. For the *differentiation score*, we subtracted the number of responses to the non-reinforced odorant (CS−) from the number of responses to the CS+. A high score indicated individuals that learned to respond to the reinforced odorant, but also quickly learned to not respond to a non-reinforced odorant. This score provides a more controlled measure of learning success, as it takes only into account specific responses to the learned odorant.

Since the patriline of each bee was known only weeks after the end of the behavioural experiments, it was not possible to plan in advance the numbers of individuals per patriline or the number of patrilines with enough individuals for analysis (n>10). Due to the high number of patrilines eventually found in the experimental hive (n = 22) and in order to encompass the whole variability in honeybees’ responsiveness and learning performances within the hive, no drastic selection of individuals based on their response scores was performed. Thus, during the thermal responsiveness procedure, bees that started to respond at one temperature (for instance 45°C) and then failed to respond to a higher temperature (for instance 55°C) were kept in the sample. Such a responsiveness score was lower than expected for bees with this temperature sensitivity. To ensure that this did not affect the results, all analyses were also performed by attributing each bee a score based only on the first temperature they responded to (a score of 6 for bees responding to the lowest temperature, a score of 1 for bees starting to respond at the highest temperature, etc.). This analysis provided exactly the same results as the one presented in the text, showing a significant correlation between thermal responsiveness and aversive learning (ρ^2^ = 0.93, p<0.001), a significant effect of patrilines on both values (ANOVA, F_9,138_ = 4.37, p<0.001 et F_9,138_ = 3.44, p<0.001) and a significant correlation between patrilines’ responsiveness and aversive learning (ρ^2^ = 0.76, p<0.01).

Some bees showed a low thermal responsiveness score (0 or 1) and did not respond to the 65°C temperature on the first day. Previous work discarded such individuals directly on the ground that they do not respond to the US used on the next day for conditioning (Roussel et al. 2009). We chose to keep these individuals as they are part of the hive’s variability, and subjected them to the conditioning phase, so that they received CS and US stimulations exactly like all other individuals. We found that during conditioning and the repeated US stimulations, these individuals responded to the US at some trials (76% responded more than 4 times to the US during the 8 CS+ trials, n = 30), but they showed low learning performances nonetheless (see Fig. 4CD) as they perceive the US as a low intensity stimulus.

As usual in SER conditioning, a number of bees (∼20–30%) responded already at the first trial to the CS+ (spontaneous responses). While the responses of these individuals cannot unambiguously be attributed to aversive learning, these bees often show that they learned specifically the CS+, as they stop responding to the CS− in the course of training. For this reason, the analyses of the two learning scores were performed twice, once with all individuals, and once taking only into account bees that did not respond at the first CS+ trial. As detailed in the results, both analyses gave the same outcome.

At the individual level, bees were grouped by heat responsiveness score and their average learning performance scores were calculated, thus allowing a clear representation of the relationship between the two variables. Average scores ± standard error of the mean (SEM) are shown in the figures. A Spearman correlation analysis was then performed on the averaged scores. At the patriline level, bees’ thermal responsiveness and aversive learning scores were calculated per patriline and both scores were averaged for the correlation. One way ANOVA was also used to compare the variations of thermal responsiveness and aversive learning performance scores among patrilines. All data were analyzed with STATISTICA V5.5 (StatSoft, Tulsa, USA).
